# Dr. Teale’s Case of Strangulated Ventral Hernia

**Published:** 1842-11-05

**Authors:** T. P. Teale


*Strangulated Ventral Hernia.—Operation.* By Mr. T. P. Teale.—Richard Morton, æt. 69, of plethoric habit, was admitted into the Leeds General Infirmary on the 1st of June, 1842, labouring under symptoms of obstruction of the bowels.

He states, that after violent exertion about two years ago, a tumor formed at the left side of the abdomen, which has never since then entirely disappeared.

At five o'clock this morning, he began to suffer from severe pains in the abdomen, with frequent vomiting. He is now much exhausted, vomiting large quantities of black fluid, like coffee-grounds; much perspiration; pulse 118, intermittent; abdomen tender, more especially in the left iliac region, where a flattened oval tumor, about three inches in length, is perceptible; this tumor is situated beneath the aponeurosis of the external oblique, which forms a tense and smooth covering of the tumor. The bowels have not acted since yesterday. A dose of cal. and col. immediately, and ol. ricini in an hour afterwards. A large clyster to be injected.

The injection brought away a small quantity of fæcal matters, but as the vomiting and pain in the abdomen continued, Mr. Teale determined to operate.

An incision was made in the long axis of the tumor, exposing the aponeurosis of the external oblique, which was next divided, when the hernial sac, covered by a rather thick layer of tissue, presented itself. On opening the sac, a coil of large intestine, highly vascular, and a small portion of omentum were exposed. The constriction was found to consist in an opening in the internal oblique and transversalis muscles, presenting a sharp tendinous edge at its mesial border, where it was contiguous to the linea semilunaris.

The opening was dilated at its upper border, the intestine was returned into the abdomen, but the omentum, having contracted extensive adhesions, was allowed to remain in the sac.

In half an hour after the operation, the patient had a copious fæcal evacuation, and the abdomen became rather softer. Pulse 120, very feeble. On the following day he died.

*Sectio cadaveris.*—A small portion of the sigmoid flexure of the colon had again descended into the sac, where it had formed adhesions; these, however, were so lax as to allow of the gut being readily pushed up. The mucous membrane of the whole of the large intestine, from the cœcum to the protruded portion of the sigmoid flexure, was quite black and of soft consistency. The interior of the cœcum and colon contained black fluid similar to that which had been vomited ; the portion of sigmoid flexure lying in the sac was considerably injected, but its mucous membrane did not exhibit very much discoloration ; below this part the gut appeared perfectly natural. The small intestines were natural, nor was there any effusion in the peritoneal cavity, nor deposition of lymph upon the serous surface.—*Provincial Medical Journal.*

